# AtPAN: an integrated system for reconstructing transcriptional regulatory networks in *Arabidopsis thaliana*

**DOI:** 10.1186/1471-2164-13-85

**Published:** 2012-03-08

**Authors:** Yi-An Chen, Ying-Chi Wen, Wen-Chi Chang

**Affiliations:** 1 Institute of Tropical Plant Sciences, National Cheng Kung University, Tainan 701, Taiwan; 2 Institute of Bioinformatics and Biosignal Transduction, National Cheng Kung University, Tainan 701, Taiwan

## Abstract

**Background:**

Construction of transcriptional regulatory networks (TRNs) is of priority concern in systems biology. Numerous high-throughput approaches, including microarray and next-generation sequencing, are extensively adopted to examine transcriptional expression patterns on the whole-genome scale; those data are helpful in reconstructing TRNs. Identifying transcription factor binding sites (TFBSs) in a gene promoter is the initial step in elucidating the transcriptional regulation mechanism. Since transcription factors usually co-regulate a common group of genes by forming regulatory modules with similar TFBSs. Therefore, the combinatorial interactions of transcription factors must be modeled to reconstruct the gene regulatory networks.

Description For systems biology applications, this work develops a novel database called *Arabidopsis thaliana *Promoter Analysis Net (AtPAN), capable of detecting TFBSs and their corresponding transcription factors (TFs) in a promoter or a set of promoters in *Arabidopsis*. For further analysis, according to the microarray expression data and literature, the co-expressed TFs and their target genes can be retrieved from AtPAN. Additionally, proteins interacting with the co-expressed TFs are also incorporated to reconstruct co-expressed TRNs. Moreover, combinatorial TFs can be detected by the frequency of TFBSs co-occurrence in a group of gene promoters. In addition, TFBSs in the conserved regions between the two input sequences or homologous genes in *Arabidopsis *and rice are also provided in AtPAN. The output results also suggest conducting wet experiments in the future.

**Conclusions:**

The AtPAN, which has a user-friendly input/output interface and provide graphical view of the TRNs. This novel and creative resource is freely available online at http://AtPAN.itps.ncku.edu.tw/.

## Background

Appropriately regulating the gene expression in plants is essential for developing or responding to an environmental stimulus. At the transcription level, transcription factors (TFs) profoundly impact gene expression, acting either as activators or repressors by binding to the promoter of target genes (TGs). Many bioinformatics web servers, including PlantCARE [1], AthaMap [2,3] and PlantPAN [4] have adopted the analysis of transcription factors and their corresponding binding sites in the promoter of genes as their primary strategy. However, most of those servers only provide information on transcription factor binding sites (TFBSs) and neglect their corresponding transcription factors. As a valuable resource for gene regulatory studies in *Arabidopsis *(*Arabidopsis thaliana*), AGRIS [5] provides transcription factors, predicted and experimentally verified *cis*-regulatory elements (CREs) as well as their interactions. Unfortunately, AGRIS lacks an interface for customized sequence analysis. Although the above resources have big contribution in advance the knowledge about gene regulation in plants, a common problem that users encounter when using these resources is that numerous TFBSs appear in the analysis results and are confused to select candidate TFBSs for further experiments. Above developments motivate this work to develop a more accurate system to elucidate the gene transcription regulatory networks (TRNs).

Microarray high throughput method is helpful in understanding whole genome gene expression profiles. Following the development of whole-genome microarrays and the increased availability of gene expression data in *Arabidopsis*, significant efforts have been denoted to unraveling the relationships between genes, such as GeneMANIA [6] and ATTED-II [7]. Both of these approaches integrate microarray datasets and calculate the level of co-expression between each pair of genes by a mutual rank (MR) or Pearson correlation score. Although some web servers have constructed networks by co-expression analysis, inferring the underlying genetic networks by relying only on the microarray data may be inadequate evidence. Therefore, how to combine the promoter analysis with the microarray data has received considerable interest. As demonstrated previously, genes with a similar expression pattern are often enriched for similar functions [8]. Vandepoele et al. also indicated that the genes with similar *cis*-regulatory elements are typically co-expressed in microarray analysis [8]. However, to our knowledge, none of the resources incorporate microarray co-expression data of TFs and their TGs in promoter analysis system or in TRNs reconstruction system in *Arabidopsis*. Consequently, in addition to integrating microarray co-expression data in the promoter analysis system, this work attempts to provide high confident TFs that are co-expressed with their TGs.

The databases IntAct [9], BioGRID [10], and BIND [11] help to elucidate protein-protein interactions. Recently, while collecting several sets of molecular interactions data from IntAct, BioGRID, BIND, and TAIR databases, PAIR [12] has supplied 5,990 experimentally reported molecular interactions in *Arabidopsis thaliana *together with 145,494 predicted interactions, making it the most comprehensive and highly reliable database of the *Arabidopsis *interactome. Comprehensively displaying TRNs requires taking the relationship between TFs and other proteins into consideration. In addition to the promoter analysis in a single gene, Su et al. posited that the transcription factors co-regulate a common group of genes by forming regulatory modules, which consist of analogous TFBSs; in addition, TFs and genes targeted by the same TFs may have a similar expression pattern and function [13]. Hence, the combinatorial interactions of transcription factors must be modeled to reconstruct the gene regulatory networks.

This work develops a comprehensive system, AtPAN (*Arabidopsis thaliana *Promoter Analysis Net), for promoter analysis in *Arabidopsis*. Additional file 1: Figure S1 illustrates the concepts of AtPAN. These concepts are helpful in elucidating the relationship between TFs and their TGs, as well as reconstructing their TRNs. The experimental and computational prediction results are easily distinguished from the output results. Additionally, AtPAN provides a comparative analysis function to identify the TFBSs in conserved regions between promoters of homologous genes or two input sequences. Different graphical outputs depending on a user's requests can be downloaded to simply present the bioinformatics analysis results for biologists. Thus, AtPAN is an effective and user-friendly resource for analyzing gene regulation in *Arabidopsis*.

### Construction and content

**Figure 1:**
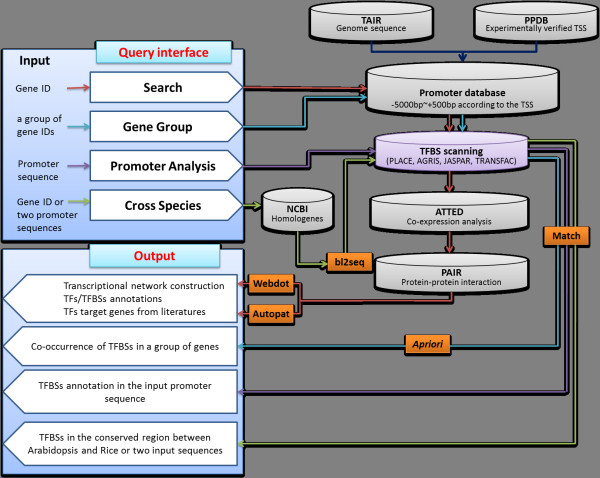
**System flow of AtPAN**. "Search", "gene group", "promoter analysis", and "cross species" are four major gateways in AtPAN. The analysis processes of these four functions are followed by different color lines displayed in the figure. "search" is denoted by a red line; "gene group" is denoted by a blue line; "promoter analysis" is denoted by a purple line; and "cross species" is denoted by a green line.

AtPAN, a web-based system, runs on an Apache web server on a Linux operation system. Contents of the integrated databases (including gene information, gene ontology (GO), gene sequence, promoter sequence, TFBSs, TFs, the information of protein-protein interaction, and gene co-expression data from microarray data) are stored in a MySQL relational database system. Also, all tables are connected by Gene ID. All web pages and data parsers are written in PHP and Perl. Figure 1 displays the system flow chart of AtPAN. The detailed methods are described as follows.

### Integrating external databases

Gene information (i.e. gene ID, gene locus, gene description, gene location, GO terms, and genomic sequence) of *Arabidopsis thaliana *were obtained from TAIR (TAIR10_genome_release) [14]. The genomic positions of 13,699 experimentally verified transcription start sites (TSSs) were downloaded from PPDB (plantpromoterdb) [15]. Addtionally, the promoter sequence of each gene in AtPAN was determined as the region from 5000 bp upstream to 500 bp downstream of the TSSs. For genes lacking positional information on the TSSs, the gene start sites recorded in TAIR database were used as the point of reference. The number of collected gene transcripts from *Arabidopsis *is 39,296. Additionally, the annotated information on the homologous genes was obtained from the NCBI homologene database [16]. The number of homologous groups of *Arabidopsis *and rice is 22,436 pairs. Moreover, the metabolic pathway maps of each genes were collected from Kyoto Encyclopedia of Genes and Genomes (KEGG) database [17]. Furthermore, the transcription factor binding profiles were collected from PLACE [18], TRANSFAC (public release 7.0) [19], AGRIS [5] and JASPAR [20]. The TFBSs and their corresponding TFs were collected manually from the literature and public resources described above. The number of TFs (which have corresponding TFBSs) used in TFBSs scanning in AtPAN is 441. To identify co-regulated TFs and their target genes, co-expression microarray data were downloaded from ATTED-II c4.1 [21]. The protein-protein interactions (PPIs) for TRNs reconstruction were accessed from PAIR [12]. The experimentally verified and computationally predicted PPIs are 5,812 and 141,605, respectively.

### Identifying TFBSs in promoter sequences and homologous conserved regions

Following determination of the promoter region, the transcription factor binding profiles were used to scan TFBSs in promoter sequences. MATCH detects the TFBSs in a promoter sequence by using the TFBS matrix from TRANSFAC public release 7.0 [19]. The default values of core similarity and matrix similarity of MATCH program were set to 0.9 and 0.8, respectively. The consensus TF binding sequences from PLACE, AGRIS and JASPAR were also used to scan TFBSs in a promoter sequence by homemade perl program. The TFBS enrichment score denotes the percentage of genes harboring this TFBS consensus sequence in their promoter. The score is calculated as the following formula: the number of genes harboring this TFBS sequence in their promoter over the total number of *Arabidopsis *genes. The lower enrichment score implies a more significant amount of the TFBS. To identify TFBSs in conserved homologous gene promoters, the homologous genes among *Arabidopsis *and rice in the cross-species analysis were extracted from NCBI. The program BLAST 2 Sequences [22] was used to define the conserved regions in each pair of homologenes. Based on the conservation of homologous promoter sequences, TFBSs within the conserved regions were identified. The identified conserved sites are more reliable than those non-conserved regions in the analyses of the transcriptional regulation in *Arabidopsis *genes.

### Identifying co-expressed TRNs of TFs and their target genes

The co-expression microarray data were used to determine high confident TFs from numerous predicted TFs in a gene promoter. The co-expression microarray data were downloaded from ATTED-II c4.1 [21]. Mutual Rank (MR) values were used to evaluate the correlation between gene A and gene B in ATTED-II. Mutual Rank (MR) is the geometric average of the Pearson's correlation coefficients (PCCs) rank from gene A to gene B and that of gene B to gene A, and it performs well to compare the coexpression strengths than PCCs in previous study (Obayashi and Kinoshita, 2009). Here, the reconstruction of co-expressed TRNs corresponded to MR values in AtPAN. If users search for the TFBSs in a gene promoter, the predicted TFBSs and their corresponding TFs appear in the results page. AtPAN then calculates MR values of each pair of gene A and predicted TFs. If the MR values of the pair of TFs and gene A exceed the setting threshold, the TFs are defined as high confident TFs. The co-expressed genes were also retrieved according to the high confident TFs. Consequently, the co-expressed TRNs can be identified. Notably, MR values were normalized from 0 to 1 since the range of MR values provided from ATTED-II is too large and not convenient for users to set the threshold in web page. The normalized MR score can be set, depending on the users. A larger score implies a higher correlation of the pair genes.

Furthermore, the TRNs of each TFs and their target genes were also determined in AtPAN. As an unsupervised pattern search method, AutoPat reconstructs the relationships between TFs and their target genes from literatures [23]. AtPAN incorporate AutoPat method to identify the networks of TFs and their target genes. Additionally, the relationships of TFs and other proteins were considered in TRNs reconstruction. The experimental and predicted PPIs data were downloaded from PAIR database and utilized in TRNs construction.

### Identifying the co-occurrence of TFs/TFBSs in a group of gene promoters

A group of gene IDs and promoter sequences is allowed to input the system in order to identify the TFs that co-regulate a group of genes. All possible TFs/TFBSs in the group of promoters were then determined. Consequently, *Apriori *is implemented to mine association rules for a group of input data [24]. A set of TFs, which bind to target sites, is assumed to participate in regulating gene transcription. Here, *Apriori *was used to discover the co-occurrence of TFs/TFBSs and combinatorial TFs/TFBSs in a group of promoter sequences. Two important parameters, *Support *and *confidence*, should be considered in utility of *Apriori*. The Support score is the frequency of promoters containing the combinatorial TF/TFBS A and B, while the confidence is the frequency of promoters containing TF/TFBS A also containing B. Following mining of the co-occurrences of TFs/TFBSs in the group of gene promoter sequences, the statistical significance of each TF/TFBS should be examined against the background set of gene promoters, based on the hypergeometric equation (*p*-value) as refer to Chang et al. [4]. After the threshold of support and confidence score by user are set, the combinatorial TFs/TFBSs that co-regulate the gene group can be identified. Furthermore, protein-protein interaction and TF-target genes network could be reconstructed. A support vector machine (SVM) PPIs prediction model was provided from PAIR. The edge of PPIs map was determined using the SVM score. To extend the efficacy of "Gene Group" analysis of AtPAN, the SVM score can be set by users to identify various TRNs, depending on how large the edges of interested.

### Graphical visualization and table list

The regulatory features located in the promoters were presented graphically or tabulated. A graphical interface was implemented using the GD library of a PHP programming language. Graphviz is a graph visualization software for drawing TRNs in AtPAN. Following completion of the analysis, numerous regulatory characteristics, including TFBSs, PPIs, and the relationships between TFs and TGs, are both shown in a graph and a table.

### Utility

#### Web interface of the AtPAN system

AtPAN is a web-based system that can thoroughly identify co-expression TRNs, including protein-protein interaction and relationship of TFs-TGs in *Arabidopsis*. AtPAN allows user queries based on gene ID, locus, keywords and sequence. After promoter extraction, the system can identify efficiently the TRNs incorporated with co-expression microarray data. The TFBSs within the conserved regions of homologous genes can also be determined. Moreover, the combinatorial TFs/TFBSs can be identified in a group of gene promoter sequences. The protein-protein interaction and TFs-TGs TRNs of a group of genes can be retrieved. The web interface of AtPAN is illustrated briefly as follows.

**Figure 2:**
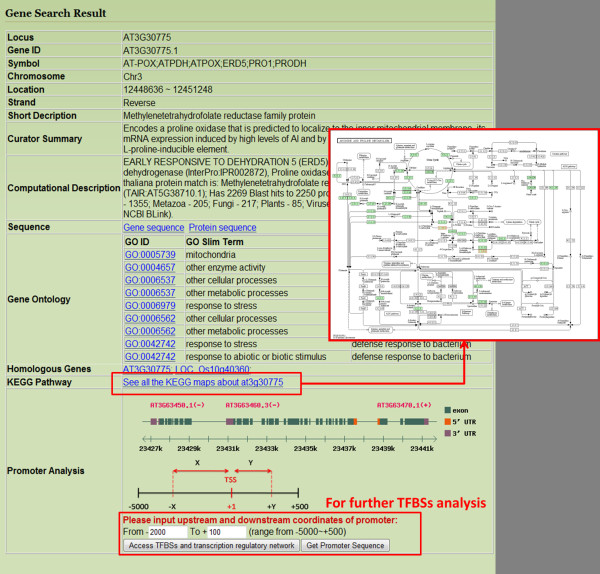
**Search result interface of AtPAN**. A table displays the general information of the query gene. The related metabolic pathways are hyperlinked. Further analysis of TFs/TFBS can be retrieved in the bottom of the resultpage.

**Figure 3:**
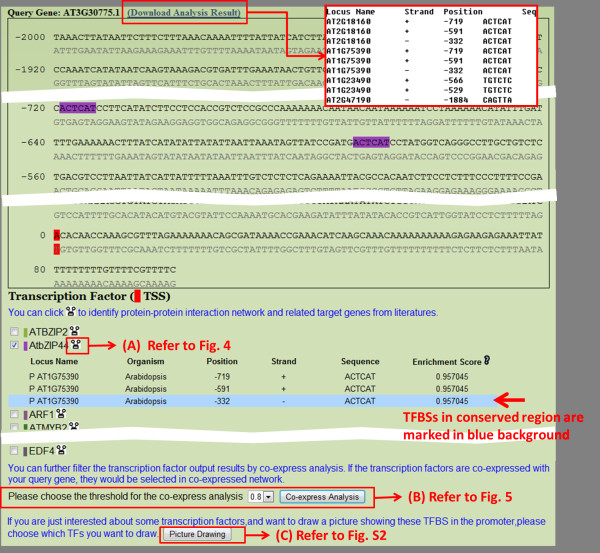
**TFs/TFBSs scanning results of AtPAN**. TF/TFBSs results and three further analysis functions are displayed. Three further analysis functions are identifying TFs-protein and TFs-TGs interaction networks of each identified TFs (A), identifying co-expressed TFs and query gene TRNs (B), as well as providing a TFBSs drawing tool for biologists (C). The blue background marked in the TFBSs sites refers to the sites in the conserved regions between homologous gene promoters.

**Figure 4:**
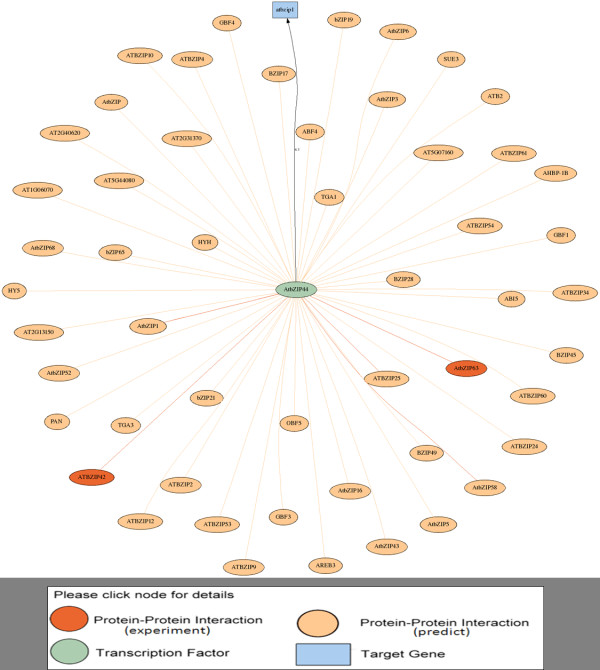
**Illustrative example of TFs-protein and TFs-TGs interaction networks**. The darkish orange, light orange, green, and blue colors of the nodes denote the experimental PPIs, computational predicted PPIs, TFs, and TG, respectively.

**Figure 5:**
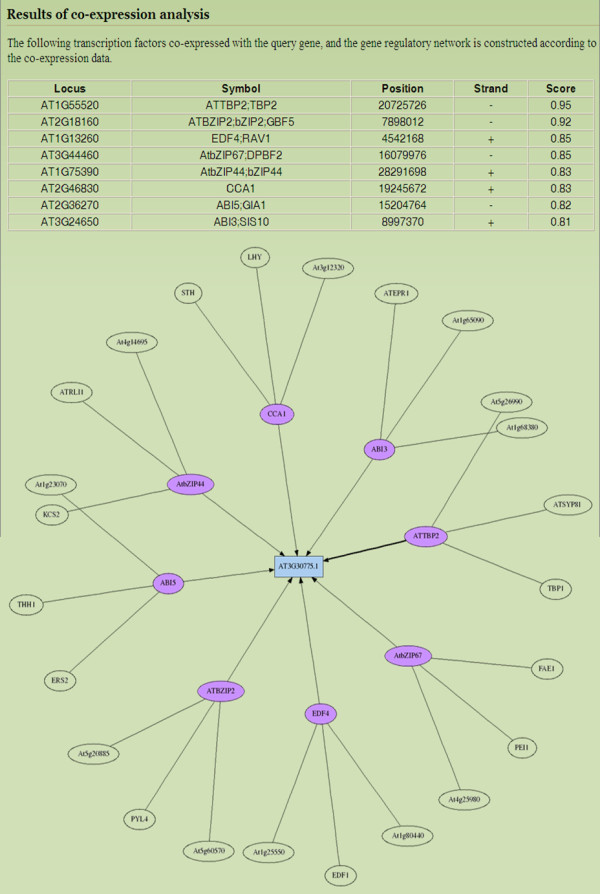
**Illustrative example of co-expressed TRNs in AtPAN**. This figure reveals that indicates AtZIP TFs are involved in ProDH (At3G30775.1) co-expressed TRNs. The purple nodes are TFs, which co-express with the target gene (blue node). The limpid nodes with black line are proteins, which interact with the TFs (purple nodes).

**Figure 6:**
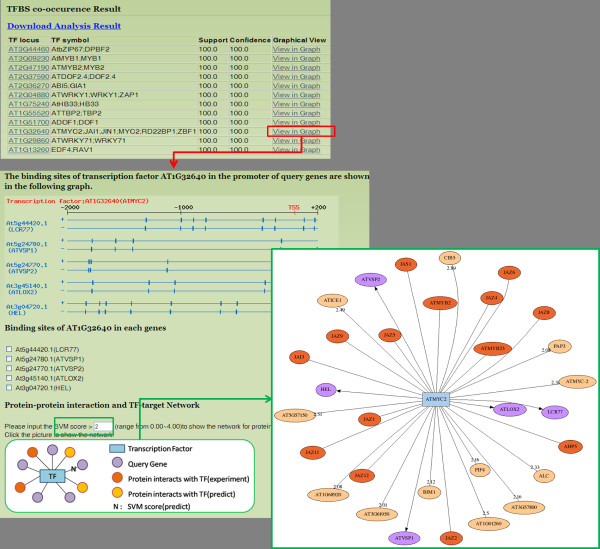
**Output interface of "Gene Group" analysis in AtPAN**. Co-occurrence of TF/TFBSs is listed in a table and illustrated in a diagram. The TRN of the group of genes is displayed in a new window. This example reveals that AtMYC2 (shown in blue node) regulates several JA-mediated plant defense genes expressions such as LCR77, AtLOX2, HEL, AtVSP1, and AtVSP2 (shown in purple nodes). Furthermore, AtMYC2 cooperates with JAZ proteins in the plant defense pathway. The PPIs data from experiments and computational prediction are shown in darkish and light orange, respectively.

The major functions of AtPAN are to identify high confidence TFs/TFBSs in promoter sequences and reconstruct TRNs in *Arabidopsis*. Four gateways in the AtPAN web interface are search, gene group, promoter analysis, and cross species. In the search gateway, a gene IDs, locus and keywords are allowed for further analysis. Figure 2 displays the interface of output results of the search function. The interface contains general information such as gene symbol, description, chromosome location, and gene ontology (GO) of the input gene. Moreover, the metabolic pathways related to the search genes can be retrieved in AtPAN. AtPAN also provides a flexible interface to obtain gene, protein and promoter sequences. At the bottom of the result page, a button is designed to further analyze TFs/TFBSs in the promoter region and TRNs reconstruction. Figure 3 summarizes the TF/TFBSs scanning results of AtPAN. This result page displays TF/TFBSs results and three further analysis functions. First, the page lists the TFs identified in the promoter region, in which TFBSs are marked in different colors according to the sequence. If the TFBSs are determined in the conserved regions between *Arabidopsis *and rice, the sites information is marked in a blue background (Figure 3). Consequently, three further analysis functions are identifying TFs-protein and TFs-TGs interaction networks of each identified TFs, identifying co-expressed TFs and query gene TRNs, and providing a TFBSs drawing tool for biologists. A miniature figure besides each TF can be clicked for TFs-protein and TFs-TGs interaction networks. Figure 4 displays an example of the output interface. Additionally, various co-express analysis thresholds can be set to reconstruct the co-expressed TRNs. The expression profiles of TFs that are co-expressed with the query gene can be figured out together with all co-expressed genes in a TRN. Figure 5 illustrates such an example. Finally, a special tool developed by AtPAN is "TFBSs drawing tool". Some users always require schematic diagrams of promoter analysis results for presentation. Here, users can select the TFBSs that they want to display. Additional file 1: Figure S2 shows the automatic generation of a schematic diagram. The gene group analysis function shows a group of *Arabidopsis *IDs that are allowed to input into the system, co-occurrence of one TF/TFBS and combinatorial TFs/TFBSs in a set of promoters then be obtained. The protein-protein interaction and TFs-TGs TRNs of this group of genes can be retrieved by further analysis in AtPAN (Figure 6). Moreover, the function of promoter analysis resembles that of search. Differently, a promoter sequence is required and TFBSs profiles from *Arabidopsis *or all plants can be used to identify TFBSs in the query sequence. Additionally, the cross species gateway function can conveniently analyze TF/TFBSs in the conserved regions between homologous gene promoters by the direct input of *Arabidopsis *Gene ID or two promoter sequences in the FASTA format. Following processing of the input data, the paired sequences are displayed in distinct colors to distinguish the conserved regions from the non-conserved regions. Additional file 1: Figure S3 displays the TFs/TFBSs in the conserved regions.

## Discussion

### Case study of reconstruction of co-expressed TRNs in *Arabidopsis*

Following the development of DNA microarrays and next generation sequencing (NGS) in experimental biology, scientists have adopted these technologies to study transcriptome regulation in a genomic wide scale in numerous species. Fukushima et al. thoroughly explored the feasibility of applying high-throughput omics approaches to reconstruct TRNs in plant systems biology [25]. Furthermore, Saito et al. indicated that transcriptome co-expression analysis is critical for studying the functional genomics of plants [26]. Therefore, AtPAN provides an effective platform to reconstruct co-expressed TRNs of *Arabidopsis *genes. This study used Proline Dehydrogenase (ProDH, AT3G30775.1) to display this efficient system. As is well known, proline accumulation occurs in response to stresses such as dehydration, cold, and salinity [27]. However, this accumulation is reversible, and proline contents return to basal levels when the stress is released [28,29]. ProDH is a major enzyme for proline degradation; ProDH should be repressed under a stress environment. Although dehydration represses the expression of ProDH, induction occurs during rehydration. A recent work demonstrated that the ACTCAT sequence in ProDH promoter regions is essential to response ProDH induction [27]. Satoh et al. also indicates a subgroup of the basic domain/leucine zipper (bZIP) TFs is involved in ProDH gene activation in *Arabidopsis *[27]. Therefore, whether AtPAN can discover the relationship between bZIP TFs and ProDH gene expression should be determined. As expected, some AtbZIP TFs such as AtbZIP44 and AtbZIP2 mentioned in Satoh et al. can be identified in co-expressed TRNs (Figure 5). Obviously, several *cis*-acting elements, including ACTCAT (the TFBSs of AtbZIP44 and AtbZIP2), are retrieved by AtPAN (Additional file 1: Figure S4). This figure reveals a TFBS located in -312 bp from TSS, which is in the conserved region between homologous gene promoters and may be important for ProDH gene regulation in prospective works. Additionally, it is known that ABA is required for proline accumulation during a stress response, however, ABI is a negative regulator for proline accumulation in *Arabidopsis *[30]. Based on results of our analysis, two ABI TFs are identified near the TSS of ProDH (Additional file 1: Figure S4). This finding might suggest that ProDH promoter is not only regulated by bZIP TFs, but also by ABI during stress response. It may give a new direction of an experimental research on ProDH in the future. Accordingly, the analysis results of AtPAN can help to identify the TRNs of *Arabidopsis *genes.

### Case study of reconstruction of TFs-TGs networks in gene group analysis

As is well known, secondary metabolites play critical roles in plant defense systems such as the Jasmonic acid (JA) signaling pathway during insect herbivores [31,32]. JA responses to insect herbivore damage and triggers many proteins involved in plant defense. First, JA conjugates to an amino acid and binds to SCF^COl1 ^protein complex. This complex then targets a transcription repressor, JAZ, which blocks MYC2 transcription factor activity. Consequently, the binding leads polyubiquitination of JAZ and subsequently degrades JAZ, while MYC2 can activate plant defensive genes [31,32]. Pauw and Memelink reviewed a situation in which PDF1.2 (also known as LCR77, At5g44420), PR4 (also known as HEL, At3g04720), VSP1 (At5g24780), VSP2 (At5g24770), and LOX2 (At3g45140) are involved in a JA-mediated defense system, and regulated by AtMYC2 transcription factors [33]. In order to evaluate the performance of AtPAN in plant systems biology research, these five gene IDs (i.e. At5g44420.1, At5g24780.1, At5g24770.1, At3g45140.1, and At3g04720.1) are input into the gene group analysis in AtPAN. Following the analysis processes, AtMYC2 transcription factors are retrieved from AtPAN, this analysis result indicates that AtMYC2 is co-occurrence in these five gene promoters (Figure 6). Our results further demonstrate that AtMYC2 bind to JAZ proteins with respect to the results of protein-protein interaction and the TFs-TGs target network (SVM score was set 2.0) (Figure 6). The analysis results are entirely consistent with those reported for plant defense mechanisms in a previous work [33]. Furthermore, both AP2-domain family TFs ORCA2 and ORCA3 are induced by JA and regulate the downstream gene expression in *C. roseus *[34,35]. Lorenzo et al. indicated that *Arabidopsis *ERF1 is an AP2-domain family transcription factor and regulates JA and ethylene response genes, including PDF1.2 (LCR77) and PR4 (HEL) [36]. Additional file 1: Figure S5 displays the same results in TRNs analysis. Moreover, according to previous studies, JA in *C. roseus *induced numerous helix-loop-helix (bHLH) TFs [37]. AtMYC2 is also one of the bHLH TFs in *Arabidopsis*. However, in previous work, no other bHLH TFs were identified in the JA responsive pathway during the plant defense mechanism, as well as AtMYC. According to our TRNs analysis results (Figure 6), several bHLH TFs such as ATICE1, CIB5, AT5G57150.1, AT1G68920.1, AT3G61950.1, BIM1, AT1G01260.1, AT3G57800.1, and ALC are predicted; most of them are novel and no related plant defense studies have been undertaken. Above results suggest that good candidates are involved in the JA-mediated plant defense system for further experiments. Furthermore, in addition to retrieving TRNs of a group of genes efficiently, the AtPAN system also offers some candidate genes for future research.

## Conlusions

This work develops a novel database called *Arabidopsis thaliana *Promoter Analysis Net (AtPAN) for analyzing high-confident TFs in a gene promoter and reconstructing co-expressed TRNs of one gene or a group of genes in *Arabidopsis*. Despite the availability of many resources such as PlantPAN, AGRIS, AthaMap, PLACE, and PlantCARE for promoter analysis in plants, those resources do not provide high-confident TFs and co-expressed TRNs of the query genes. Additionally, information of co-expression microarray data and PPIs is first incorporated to conduct promoter analysis in plants. Based on the validation results, AtPAN is effective in constructing the regulation networks in a well-known system as well as the above case study which mentioned JA-mediate plant defense system. Therefore, based on the analysis results, we recommend undertaking wet experiments in the future. A more user-friendly web interface for biologists can be provided with less complex tables and parameters on the web. Importantly, a TFBSs drawing tool is designed for users to select which TFBSs they want to display in their data presentations. Nevertheless, despite its advantage, the proposed AtPAN database has certain limitations. In this version of AtPAN, the reconstruction of co-expressed TRNs is only provided in *Arabidopsis*. Additionally, the TRN is too complex for actual plants. For instance, microRNA is also important in gene regulation and functions as a converse mechanism as TFs do. Owing to that several TFs could be discovered in TRNs of AtPAN analysis results, whether the TFs are negatively regulated by some microRNA should be determined. Therefore, efforts are underway in our laboratory to expand upon the methods used in AtPAN to other plant species such as rice, maize, and *Physcomitrella patens*. Furthermore, the relationship between microRNA and their target genes will be incorporated into AtPAN in the near future.

### Availability and requirements

The AtPAN will be continuously maintained and updated according to the increasing number of plant TFs/TFBSs. This novel and creative resource is now freely available at http://AtPAN.itps.ncku.edu.tw/.

## Authors' contributions

WCC conceived and supervised this project, and wrote the manuscript. YAC and YCW were responsible for the design, computational analyses, web interface development, and implementation of the database. All authors read and approved the final manuscript.

## Supplementary Material

Additional file 1 **Figure S1**. The concepts of AtPAN. **Figure S2**. Output example of "TFBSs drawing tool" in AtPAN. **Figure S3**. Output example of "Cross Species" analysis in AtPAN. **Figure S4**. TFBSs of AtbZIP2, AtbZIP44, and EDF4 identified in ProDG (At3G30775.1) promoter region. The blue background indicates that the TFBSs are discovered in the conserved region between homologous gene promoters. **Figure S5**. Promoter and TRNs analysis results of PDF1.2 (LCR77) and PR4 (HEL) by AtPAN. (**A**)AtERF1 co-occur in both promoter sequences; in addition, the TFBSs are displayed. (**B**) co-expression TRNs of PR4 genes, both MYC2 and ERF1 are identified in the network. Click here for file
