# Supplementary material for: AtPAN: an integrated system for reconstructing transcriptional regulatory networks in Arabidopsis thaliana
Source: BMC Genomics. 2012 Mar 8;13:85. doi: 10.1186/1471-2164-13-85 (PMC3314555; doi:10.1186/1471-2164-13-85)
Supplement: Additional file 1 — Figure S1. The concepts of AtPAN. Figure S2. Output example of "TFBSs drawing tool" in AtPAN. Figure S3. Output example of "Cross Species" analysis in AtPAN. Figure S4. TFBSs of AtbZIP2, AtbZIP44, and EDF4 identified in ProDG (At3G30775.1) promoter region. The blue background indicates that the TFBSs are discovered in the conserved region between homologous gene promoters. Figure S5. Promoter and TRNs analysis results of PDF1.2 (LCR77) and PR4 (HEL) by AtPAN. (A)AtERF1 co-occur in both promoter sequences; in addition, the TFBSs are displayed. (B) co-expression TRNs of PR4 genes, both MYC2 and ERF1 are identified in the network. [file 1471-2164-13-85-S1.PDF]

Additional file 1

Supplemental figures

Figure S1 The concepts of AtPAN

Figure S2 Output example of "TFBSs drawing tool" in AtPAN

Figure S3 Output example of "Cross Species" analysis in AtPAN

Figure S4 TFBSs of AtbZIP2, AtbZIP44, and EDF4 identified in ProDG (At3G30775.1) promoter region. The blue background indicates that the TFBSs are discovered in the conserved region between homologous gene promoters.

Figure S5 Promoter and TRNs analysis results of PDF1.2 (LCR77) and PR4 (HEL) by AtPAN. (A)AtERF1 co-occur in both promoter sequences; in addition, the TFBSs are displayed. (B) co-expression TRNs of PR4 genes, both MYC2 and ERF1 are identified in the network.

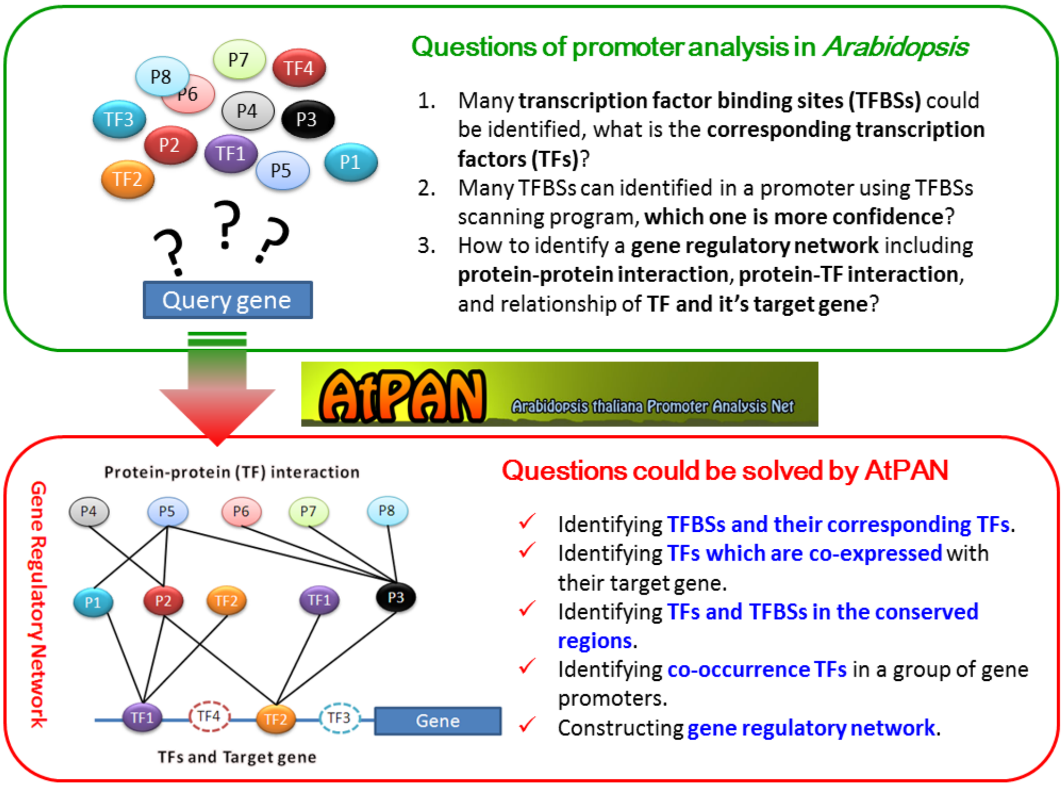

Figure S1 The concepts of AtPAN.

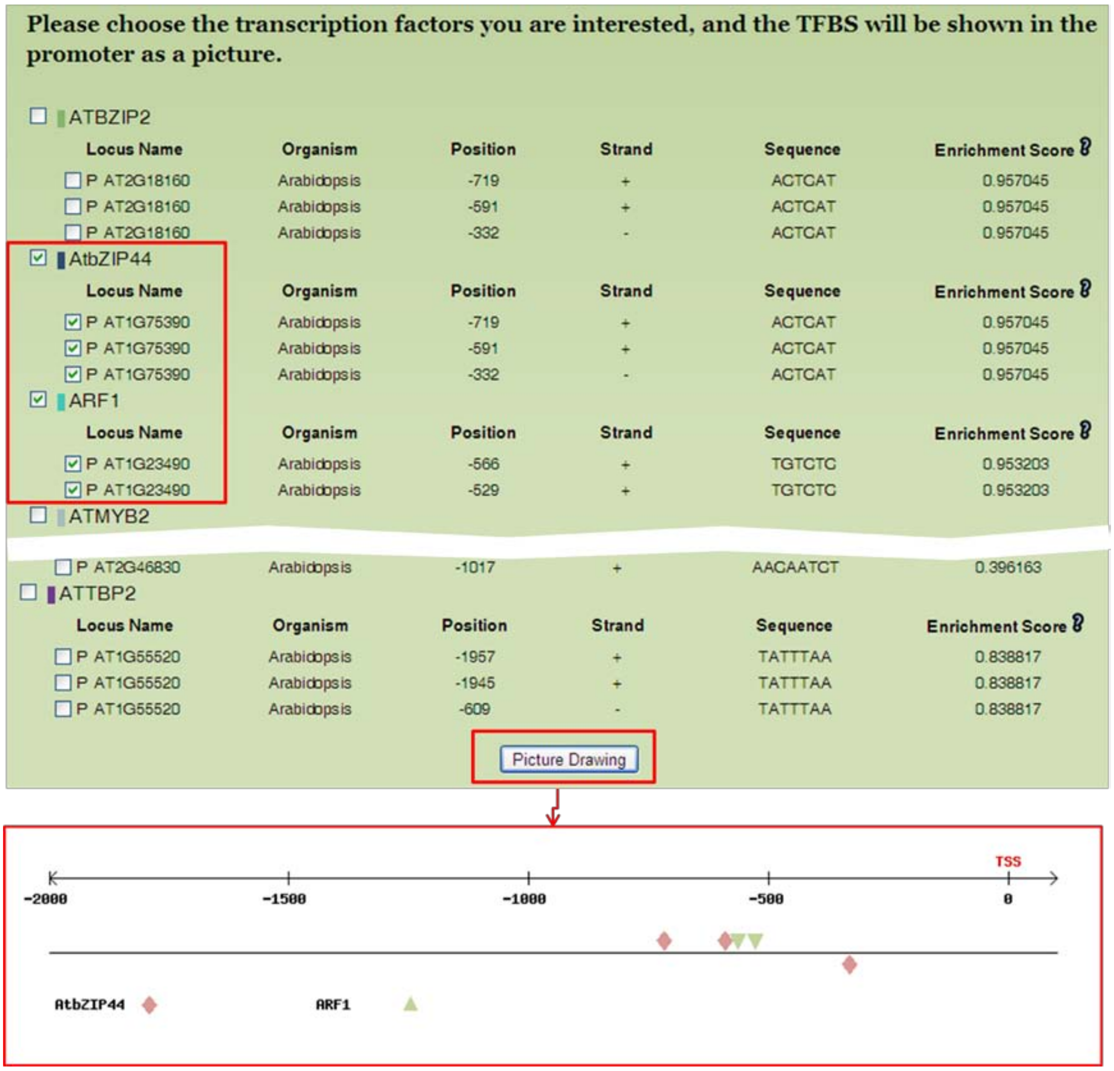

Figure S2 An output example of “TFBSs drawing tool” in AtPAN.

Conserved region between Arabidopsis and Rice promoter

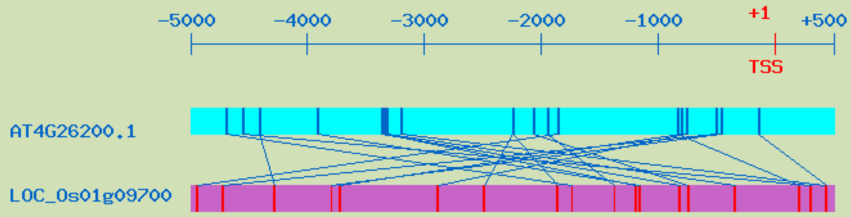

TFBS in conserved region

TF locus	TF Symbol	TFBS position	Strand	TF binding motif
AT1G51700	ADOF1;DOF1	-2239	-	AAAG
AT2G37590	ATDOF2.4;DOF2.4	-2239	-	AAAG
AT1G75240	AtHB33;HB33	-3343	-	ATTA
AT1G32640	ATMYC2;JAI1;JIN1;MYC2;RD22BP1;ZBF1	-1846	-	CACATG
AT2G36270	ABI5;GIA1	-1846	-	ACACATG
AT3G44460	AtbZIP67;DPBF2	-1846	-	ACACATG
AT1G32640	ATMYC2;JAI1;JIN1;MYC2;RD22BP1;ZBF1	-4409	+	CAATTG
AT1G32640	ATMYC2;JAI1;JIN1;MYC2;RD22BP1;ZBF1	-4409	-	CAATTG
AT1G13260	EDF4;RAV1	-471	+	CAACA
AT1G75240	AtHB33;HB33	-467	+	ATTA
AT1G75240	AtHB33;HB33	-4700	+	ATTA
AT1G75240	AtHB33;HB33	-4698	-	ATTA
AT1G75240	AtHB33;HB33	-4696	+	ATTA
AT1G51700	ADOF1;DOF1	-1944	-	AAAG
AT1G75240	AtHB33;HB33	-1938	-	ATTA
AT2G37590	ATDOF2.4;DOF2.4	-1944	-	AAAG
AT3G24650	ABI3;SIS10	-3904	-	CATGCA
AT1G75240	AtHB33;HB33	-3363	-	ATTA
AT1G29860	ATWRKY71;WRKY71	-2058	-	TGAC
AT1G75240	AtHB33;HB33	-2067	-	ATTA
AT1G75240	AtHB33;HB33	-832	-	ATTA

Figure S3 An output example of “Cross Species” analysis in AtPAN.

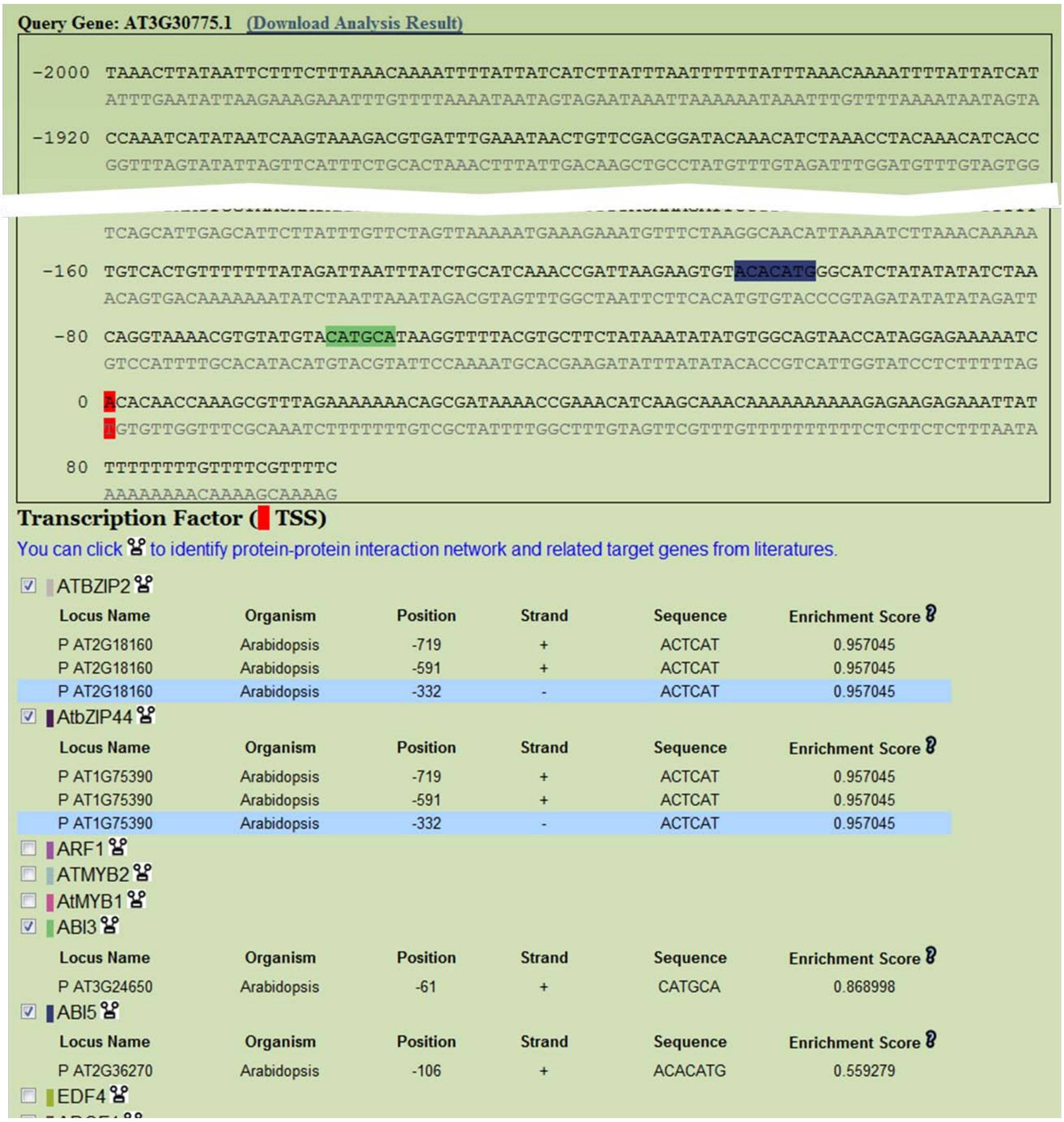

Figure S4 The TFBSs of AtbZIP2, AtbZIP44, ABI3, and ABI5 are identified in ProDG (At3G30775.1) promoter region, respectively. The blue background indicates the TFBSs are discovered in the conserved region between homologous gene promoters.

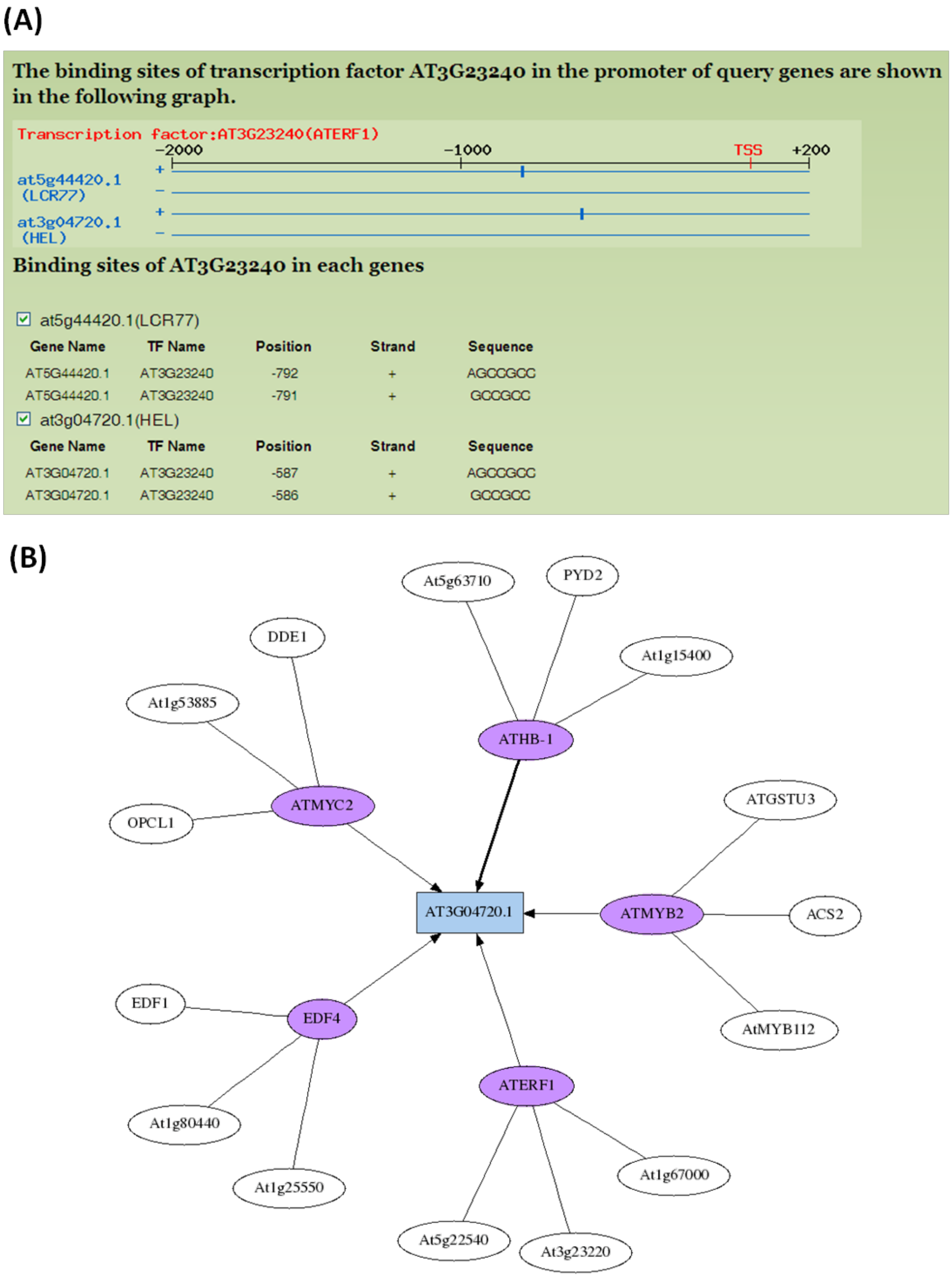

Figure S5 Promoter and TRNs analysis results of PDF1.2 (LCR77) and PR4 (HEL) by AtPAN. (A)AtERF1 co-occur in both promoter sequences, and the TFBSs are displayed. (B) co-expression TRNs of PR4 genes, both MYC2 and ERF1 are identified in the network.
